# p-BioSPRE - p-medicine Biospecimen Search and Project Request Engine

**DOI:** 10.1186/2043-9113-5-S1-S20

**Published:** 2015-05-22

**Authors:** Christina Schröder, Tim Jüttner, Matthias Dobkowicz

**Affiliations:** 1 Fraunhofer IZI-BB, 14476 Potsdam, Germany

## Characterisation

Web-based tool, networking of biobanks for personalized medicine, metabiobank, provision of trans-institutional and trans-national access to biobanks.

## Description

**Figure 1:**
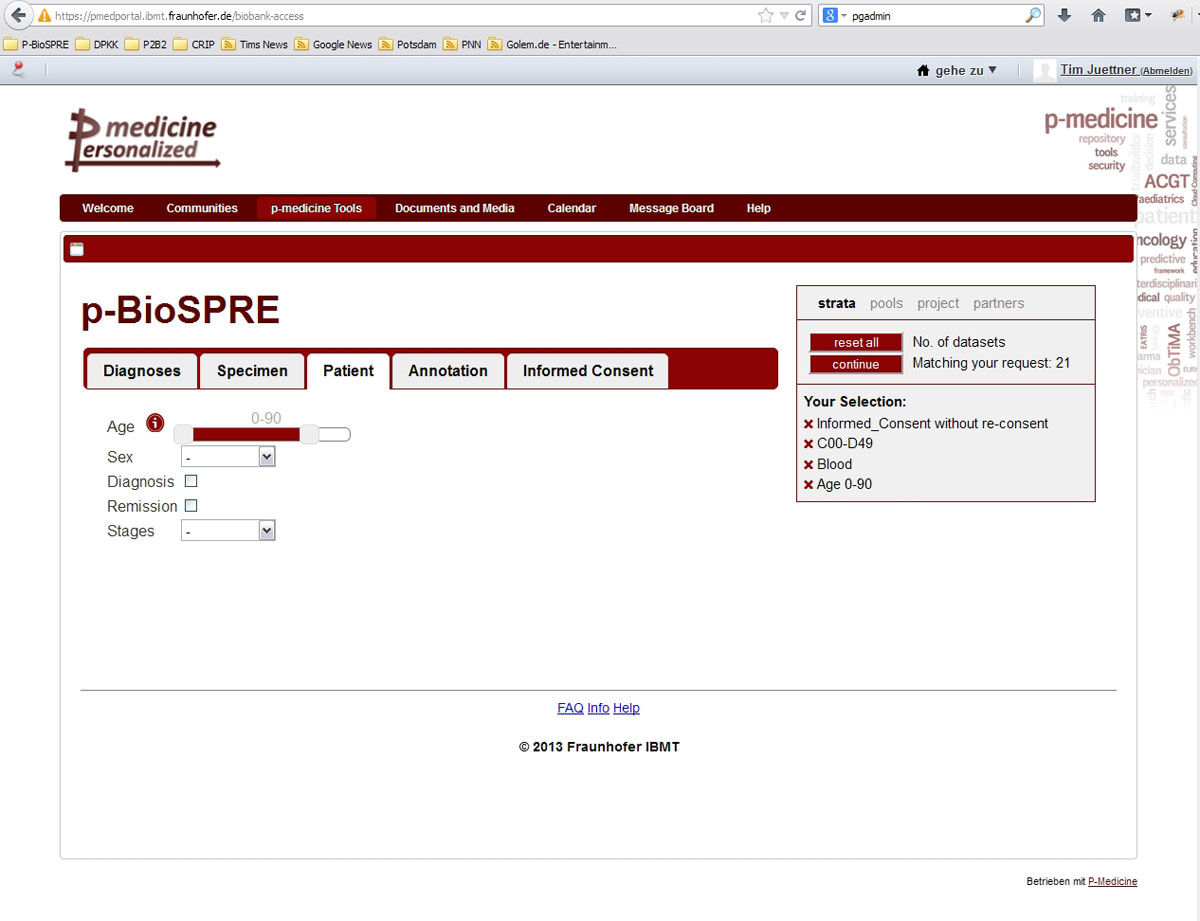
p-BioSPRE user interface displaying as example patients’ age selection

p-BioSPRE is a metabiobank providing trans-institutional and transnational access to biobanks (Figure 1) while safeguarding patients’ privacy and full biobank autonomy [1].

The p-BioSPRE architecture is based on modular CRIP tools and concepts (a metadata-driven, scalable, and robust system of relational databases with an xml-based query interface) [2]. It mirrors the hub-and-spokes structure proposed by BBMRI for national and international biobank networks. Contrary to biobank registries and catalogs, p-BioSPRE allows for up-to-date queries on a case-by-case and specimen-by-specimen basis. It caters to both: researchers looking for well-annotated human specimens from all disease areas and biobank operators conveniently offering donated specimens and data for research.

p-BioSPRE is in line with the BBMRI requirements for data integration systems and the p-medicine security framework. Its central infrastructure is maintained by Fraunhofer.

## Status of development

Access to the operative version of p-BioSPRE is restricted to p-medicine partners. The test and demo version 0.6 is on-line (links below) since May 23, 2014. Evaluation of p-BioSPRE by p-medicine clinical partners is ongoing until 2015.

## Users

Researchers looking for stratified human specimens.

Biobank operators looking for projects and partners to make use of donated specimens and data.

## Links

p-BioSPRE Demonstrator [https://preview-crip.fraunhofer.de/intern/demo/searchtool/search/p-biospre.cgi]; CRIP Toolbox [http://www.crip.fraunhofer.de/en/toolbox]
